# One-year functional and structural results of faricimab for treatment-naïve neovascular age related macular degeneration: An OCT angiography study

**DOI:** 10.1007/s00417-025-06849-y

**Published:** 2025-05-06

**Authors:** Alessandra Scampoli, Matteo Mario Carlà, Giulia Grieco, Lorenzo Governatori, Roberta Catalani, Stanislao Rizzo, Tomaso Caporossi

**Affiliations:** 1 Vitreoretinal Surgery Unit, Isola Tiberina Hospital - Gemelli Isola, Rome, Italy; 2 Ophthalmology Department, “Fondazione Policlinico Universitario A. Gemelli, IRCCS”, 00168 Rome, Italy; 3 Ophthalmology Department, Catholic University “Sacro Cuore”, Rome, Italy

**Keywords:** Age-related macular degeneration, Anti-vascular endothelial growth factor, Faricimab, Treatment-naïve, Treat-and-extend, OCT angiography

## Abstract

**Purpose:**

To explore the 1-year functional and anatomic outcomes of treatment-naïve neovascular age-related macular degeneration (nAMD) eyes undergoing a treat and extend (TAE) regimen with faricimab,

**Methods:**

Prospective interventional study on 33 eyes with treatment-naïve nAMD undergoing a loading phase of 4 monthly faricimab followed by a TAE regimen. Participants underwent functional assessment and retinal imaging with optical coherence tomography and angiography (OCT/OCTA), at baseline and follow-up visits (V0-V5). Primary outcomes were safety, changes in best-corrected visual acuity (BCVA) and central subfield thickness (CST). Secondary outcomes included changes in OCT biomarkers (intraretinal and subretinal fluid [IRF and SRF], maximum pigment epithelium detachment [PED] height) and vascular densities (VD) in the superficial (SCP) and deep capillary plexuses (DCP).

**Results:**

Mean follow-up was 14.1 ± 2.7 months. At the final visit, 36.4% eyes were on a q16w regimen, and 36.4% on q12w. Results showed significant reductions in CST (V0: 334 ± 102 μm, V5: 227 ± 47 μm, *p* < 0.001), presence of IRF/SRF and PED height. BCVA improved significantly from 0.51 ± 0.23 to 0.36 ± 0.26 LogMAR (*p* = 0.03). A dry macula after the loading phase was achieved in 63.7% of cases and correlated with longer inter-injections intervals during TAE. SCP’s VD showed a transient decrease in V1-V3 but returned to baseline values at V5, while no significant changes were observed in DCP VD. No cases of intraocular inflammation or adverse events were observed.

**Conclusion:**

Faricimab showed favorable results in treatment-naive nAMD, leading to structural and functional improvements and allowing for extended treatment intervals even in real-world setting.

## Key messages

***What is known***

• In clinical trials, faricimab showed the potential to prolong the interval between two successive injections to 16 weeks in almost 50% of the eyes in nAMD.

***What is new***

• In this real world study, a substantial proportion of eyes (36.4%) achieved a 16-week treatment interval, and another 36.4% reached a 12-week interval after more than 1 year of follow-up.

• OCT structural parameters improved during faricimab treatment, with CST, IRF, SRF and maximum PED height reduction. Additionally, 54.5% of eyes showed visual improvements during the study period.

• OCTA data are still controversial, highlighting a transient reduction in SCP’s VD, returning to baseline within the end of the study.

## Introduction

For more than ten years, anti-vascular endothelial growth factor (VEGF) medicines have been the gold standard of care for exudative neovascular age-related macular degeneration (nAMD). However, due to the recurrence of exudation, patients need repeated anti-VEGF injections, which puts a significant strain on both patients and doctors. In an effort to decrease the frequency injections while preserving visual acuity, treat-and-extend (TAE) regimens have been established [1, 2]. However, for the long-term treatment of this condition, some individuals still need periodic injections [3, 4]. The research in the last years thus focused on the development of new long-lasting therapies, particularly for resistant AMD patients.

In January 2022, the FDA approved Faricimab (Vabysmo, Roche/Genentech, Basel, Switzerland), a novel VEGF/angiopoietin-2 (Ang-2) bispecific agent for the treatment of nAMD and diabetic macular edema (DME). Ang-2 is a key regulator during angiogenesis by the negative feedback of angiopoietin 1/tyrosine kinase with immunoglobulin (Ig) and epidermal growth factor (Tie) 2 signaling. The dual action of anti-VEGF/Ang-2 aims to reduce retinal inflammation and neovascularization and to enhance vascular stability [5, 6]. In fact, faricimab injections given at 8- to 16-week intervals were shown to be non-inferior to aflibercept injections given at 8-week intervals in treatment-naive nAMD eyes in the pivotal TENAYA and LUCERNE trials [7]. Moreover, faricimab showed the potential to prolong the interval between two successive injections to 16 weeks in almost 50% of the eyes [6]. Initial findings from real-world trials have corroborated the effectiveness of faricimab, indicating enhanced retinal fluid management and visual stability in eyes affected by nAMD and DME [8, 9].

However, to date few reports analyzed the outcomes of faricimab for treatment-naïve nAMD in real world settings after a mid-term follow-up [10, 11]. In the current research, we evaluated the 1-year functional and structural outcomes of loading phase treatment followed by maintenance therapy employing a TAE regimen in 33 eyes with treatment-naïve nAMD.

## Materials and methods

This prospective monocentric interventional research, conducted at Ospedale Isola Tiberina-Gemelli-Isola, Rome, Italy included patients recruited between July 2023 and November 2023, with data collection completed by November 2024. All patients completed at least 12 months of follow-up**.** All procedures performed were in accordance with the tenets of Helsinki declaration and the investigation received the approval of the Catholic University of the Sacred Heart Ethical Committee. A complete explanation of the study protocol was provided and informed consent was collected from all study participants.

Age and sex were recorded at baseline, and each study eye underwent multimodal retinal imaging, including spectral-domain optical coherence tomography (SD-OCT) and OCT angiography (OCTA), fluorescein angiography and indocyanine green angiography, to phenotype the macular neovascularization (MNV). At each visit, patients underwent slit-lamp assessment with dilated fundoscopy, intraocular pressure (IOP), and best corrected visual acuity (BCVA). Possible adverse events were analyzed and described. During follow-ups, OCT/OCTA analysis were performed again at each time point. For statistical purposes, we selected 5 timepoints for the analysis of morpho-functional parameters: V0, baseline; V1, at the end of loading dose (week 12); V2, one month after the end of the loading dose (week 16); V3, at the first follow-up after the first extension; V4, at the one-year follow up visit (week 48 or 52); V5, at last available follow-up.

All patients received loading phase treatment in accordance with the TENAYA and LUCERNE protocols, [12] which included 4 monthly injections of 6 mg faricimab. In the maintenance phase, the injection interval was prolonged by 4 weeks upon achieving a completely dry macula; conversely, the period was reduced by 4 weeks if the condition was not met. Dry macula was characterized by the absence of intraretinal, subretinal, or sub-RPE fluid, along with either no or reduced bleeding. In this investigation, the treatment interval was established with a minimum of 8 weeks (q8w) and a maximum of 16 weeks (q16w). If a fully dry macula was not attained with the q8w regimen of faricimab injections, patients were scheduled to switch to a different anti-VEGF medication.

### OCT/OCTA data collection

OCT/OCTA imaging were performed using the RTVue XR Avanti (Optovue Inc, Fremont, California,USA). Scans with low-quality indices (< 7/10) due to significant lens opacities, frequent eyelid blinking, excessive motion artifacts, and other factors were rejected.

High-definition fovea-centered Macula Cross and HD Raster images were collected to assess for OCT biomarkers. Additionally, the RetinaCube software was used to automatically calculate the central (CMT, namely the mean retinal thickness within a 0.5-mm-radius circle at the foveal center). OCTA images were acquired using the AngioVue Retina software, automatically assessing the vascular density (VD) of the superficial and deep capillary plexuses (SCP, DCP) in the central 1-mm subfield of the Early Treatment Diabetic Retinopathy Study (ETDRS) maps. Each OCTA image underwent motion correction and 3D projection artifact removal technology to improve image quality.

The whole macula was covered by volumetric scan OCT images to assess the presence of sub-retinal fluid (SRF), intra-retinal fluid (IRF), or sub-RPE fluid. The distance between the Bruch's membrane and the sclero-choroidal boundary at the fovea was used to calculate subfoveal choroidal thickness (SFCT). At the highest point of the pigment epithelial detachment (PED), the height of the PED was measured from RPE to Bruch's membrane.

Two unbiased masked retinologists (A.S., G.G.) evaluated SD-OCT and OCT-A scans, and any disputes were settled by a third observer, T.C. The follow-up mode of the Optovue software was employed to ensure correct centration and repeatability of OCT scans. Moreover, a manual double-check was performed independently by two observers at each follow-up. When necessary, segmentation errors were manually fixed using built-in software.

The primary endpoints of this study were safety, retinal fluid resolution, mean BCVA, CST and SFCT changes over the follow-up. As secondary outcomes, we evaluated changes in OCT biomarkers and vessel densities during the treatment.

### Statistical analysis

Statistical analysis was conducted using GraphPad PRISM Software (Version 10.3; GraphPad, La Jolla, CA). The Shapiro–Wilk test was employed to assess the normality of the sample, with a p-value greater than 0.05 supporting the null hypothesis of normal distribution. Mann–Whitney U test was utilized with a 95% confidence interval (CI) to compare continuous variables. Two-way analysis of variance (ANOVA) with Dunnett correction was used to evaluate changes in BCVA and OCT parameters during follow-ups. Correlation analysis was performed using the Spearman coefficient. χ2 test and Fisher’s exact test for contingency analysis were conducted where appropriate. Mean ± standard deviation (SD) was used to present quantitative data, and a *p* < 0.05 was deemed as statistically significant.

## Results

This cohort included 33 eyes from 30 individuals with treatment-naïve AMD (24 eyes from 24 women and 9 eyes from 6 men), with an average age of 76.4 ± 7.3 years. In terms of MNV subtypes, 24 eyes (72.7%) had a type 1 MNV, 7 eyes (21.2%) a polypoidal choroidal vasculopathy (PCV) and 2 eyes (6.1%) a type 2 MNV. All eyes were followed for at least one year, with a mean follow-up of 14.1 ± 2.7 months from baseline.

**Fig. 1:**
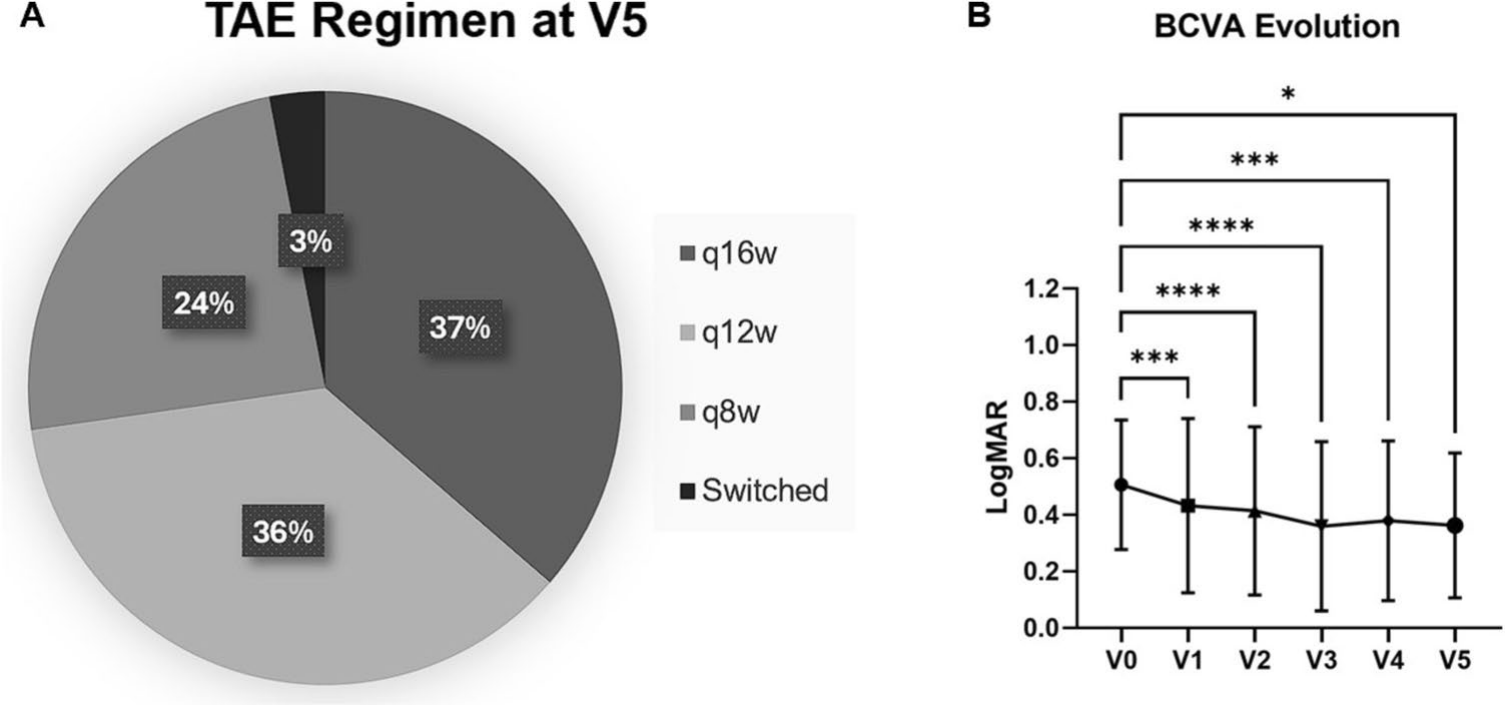
**A**) Prevalence of different intervals between injections in the treat-and-extend (TAE) regimen, at the end of the study period; **B**) Visual improvements during follow-ups. Asterisks stand for statistical significance

Mean number of intravitreal injections was 8.0 ± 1.6 per eye, with an average interval of 12.4 ± 3.2 weeks during the maintenance phase (excluding the loading phase). At the one-year follow-up visit (V4), 13 eyes (39.4%) were on a q16w regimen, 11 eyes (33.3%) on q12w, and 9 eyes (27.3%) were on q8w. At the most recent visit (V5), 12 eyes (36.4%) were on a q16w regimen, 12 eyes (36.4%) on q12w and 8 eyes (24.2%) were on q8w. Due to persistent exudative alterations after q8w faricimab injections, one eye (3.0%) was converted to intravitreal injections of aflibercept. None of the eyes showed any kind of post-injection adverse event or intraocular inflammation (IOI) (Fig. 1).

At week sixteen, one month after the end of the loading phase, 21 eyes (63.7%) showed a completely dry macula on OCT. At the one-year follow-up visit (V4), 16 eyes (48.5%) had a completely dry macula, while at the most recent appointment (V5), 18 eyes (54.5%) showed a completely dry macula. These eyes received significantly fewer injections overall throughout the course of the study period (7.2 ± 0.8 vs. 9.4 ± 0.7, *p* = 0.02). A dry macula at the end of the loading phase was associated with a longer planned injection interval at the final visit, compared to patients with residual fluid at week 16 (13.5 ± 3.4 vs. 10.3 ± 2.2, *p* = 0.03).

### Functional results

The BCVA at baseline and during follow ups were: 0.51 ± 0.23 LogMAR at V0, 0.43 ± 0.30 LogMAR at V1 (*p* = 0.002), 0.41 ± 0.29 LogMAR at V2 (*p* = 0.001), 0.36 ± 0.30 LogMAR at V3 (*p* = 0.001), 0.38 ± 0.28 LogMAR at V4 (*p* = 0.004), and 0.36 ± 0.26 at V5 (*p* = 0.03) (Fig. 1). Overall, 18/33 eyes (54.5%) showed visual improvements during the study period, In terms of functional driving vision (defined as BCVA ≤ 0.3 LogMAR), 15 eyes (45.5%) at V4 and 16 eyes (48.5%) at V5 achieved this threshold. Six eyes (18.2%) exhibited an improvement of 0.3 LogMAR or more. Conversely, 12/33 eyes (36.4%) showed stable BCVA, while 2 eyes (6.1%) showed a 0.1 LogMAR decline. The only eye showing a BCVA decline of 0.3 LogMAR had persistent exudative alterations and was switched to aflibercept. Mean visual improvement did not differ between eyes with type 1 MNV and PCV (0.15 ± 0.17 vs. 0.11 ± 0.08 LogMAR; *p* = 0.13).

A thicker CST at V0 significantly correlated with worse final visual outcomes (ρ = 0.38; *p* = 0.03). Conversely, baseline BCVA did not correlate significantly with final BCVA (ρ = 0.31; *p* = 0.16).

### Structural and microvascular evolution

During the follow-up, foveal thickness showed a significant decrease. At each time point, mean CST were, in order, 334 ± 102 μm at V0, 268 ± 86 μm at V1 (*p* = 0.002), 229 ± 48 μm at V2 (*p* < 0.001), 220 ± 37 μm at V3 (*p* < 0.001), 210 ± 38 μm at V4 (*p* < 0.001), and 227 ± 47 μm at V5 (*p* = 0.001) μm. The main drop in terms of CST was noted at the end of the loading phase (mean difference −65 μm, 95% CI 31 to 98 μm), and successively the mean CST remained pretty stable during the successive follow-ups.

Conversely, SFCT did not show significant changes during the study period (from 296 ± 102 μm at V0 to 278 ± 115 μm at V5, *p* = 0.51). As expected, eyes with PCV has thicker choroid at either V0 (424 ± 56 μm vs. 249 ± 75 μm of other MNV subtypes, *p* < 0.001) ad at final follow-up (407 ± 43 μm vs. 237 ± 57 μm of other MNV subtypes, *p* < 0.001).

In terms of fluid analysis, 21 eyes (64%) showed IRF at V0, compared to 9 eyes (27%) at V1, 3 eyes (9%) at V2 and 10 eyes (30%) at V5. Conversely, 27 eyes (82%) had SRF fluid at baseline, reducing to 15 eyes (45%) at V1, 6 eyes (18%) at V2 and 7 eyes (21%) at the end of the study period. Contingency analysis confirmed significant reduction of either IRF presence (*p* = 0.03) and SRF presence (*p* = 0.0004).

Similarly, the maximum PED height decreased from 277 ± 302 μm at V0, to 164 ± 213 μm at V1 (*p* = 0.001), 138 ± 167 μm at V2 (*p* < 0.001), 140 ± 148 μm at V3 (*p* = 0.01), 148 ± 154 μm at V4 (*p* = 0.03) and 158 ± 160 μm at V5 (*p* = 0.04). The maximum drop in terms of PED height was seen during the loading phase (mean difference −113 μm from V0 to V1, CI 26 to 199 μm), then reaching its minimum value at V2, but then showing a slight increase during the TAE regimen (+ 21 μm from V2 to V5, CI −10 to 50 μm, *p* = 0.25).

**Fig. 2:**
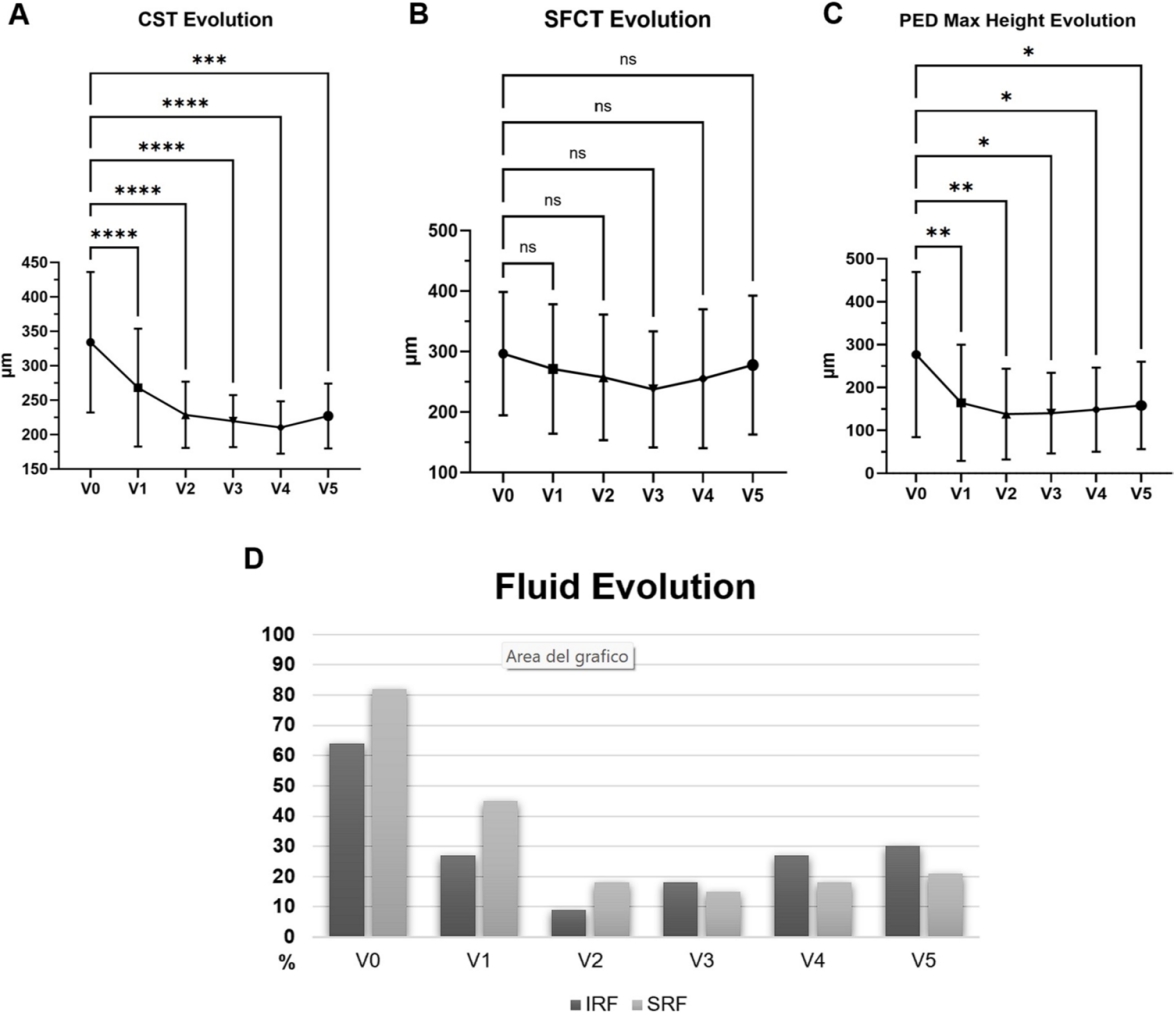
Evolution of structural parameters and OCT biomarkers. **A**) Central subfield thickness (CST) showed a significant improvement starting from V1 and being stable during the study period; **B**) Subfoveal choroidal thickness (SFCT) didn’t show significant changes connected to the antiVEGF treatment; **C**) Pigmented epithelium detachment (PED) maximum height was significantly reduced during the loading phase, reaching its nadir at V2. **D**) Prevalence of intraretinal and subretinal fluid (IRF and SRF) throughout the follow-ups, with the latter showing a better response to faricimab treatment, even if the reduction was significant for both. During the treat-and-extend regimen (V3-V5), a slight increase was highlighted in terms of IRF prevalence. Asterisks stand for statistical significance

A summary of structural parameters evolution is visible in Fig. 2.

OCTA analysis was focused on the evolution of VD in the SCP and DCP of the central ETDRS subfield. In the former, VD showed a decrease from V0 (42.8 ± 3.5%) to V1 (38.9 ± 4.4%, *p* = 0.005), V2 (36.1 ± 5.3%, *p* = 0.001) and V3 (37.0 ± 5.5%, *p* = 0.002), but went back to values comparable to baseline at V5 (41.5 ± 2.0%, *p* = 0.11). Conversely, even showing a similar evolutive pattern, no significant changes were reported in the DCP’s VD compared to baseline (47.6 ± 2.8%), in either follow-up (46.9 ± 5.1% at V1, 44.7 ± 6.9% at V2, 44.2 ± 6.6% at V3, 45.5 ± 5.2% at V4, 46.9 ± 5.4% at V5, respectively, all *p* > 0.05).

**Fig. 3:**
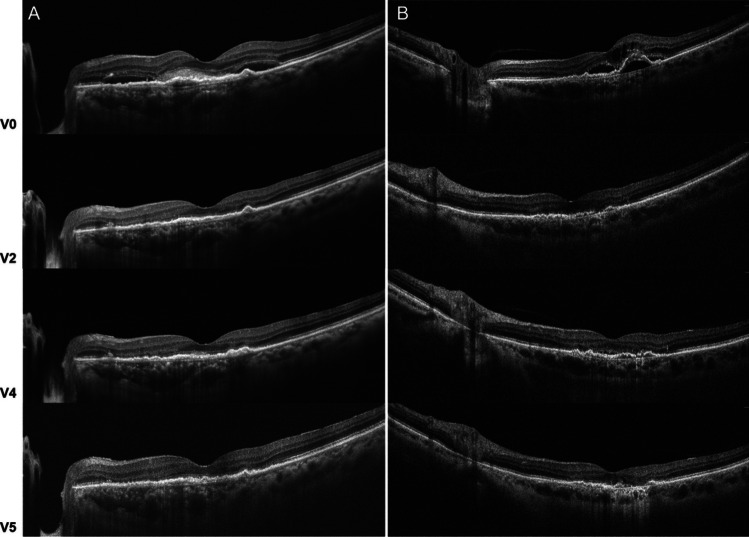
**A**/**B**) Samples of the response to the loading phase (V2) and successive treat-and-extend (TAE) regimen (V4-V5) with faricimab in 2 eyes showing a fluid-free macula at the end of the study period. However, perifoveal retinal pigmented epithelium (RPE) atrophy is noticeable in patient B at V5. In addition, in case B, residual fluid was present at V4, prompting a reduction in treatment interval from q16w to q12w, which resulted in a dry macula at V5

Samples of B-scan evolution in treatment-naïve eyes undergoing faricimab treatment are shown in Fig. 3.

## Discussion

Faricimab is a new bispecific antibody with the ability to target both VEGF and Ang-2, which has demonstrated promising results in improving vision and reducing retinal fluid in patients with nAMD. Several real-world studies, conducted globally, reported the short-term efficacy, durability, and safety of intravitreal faricimab in the treatment-naïve nAMD. In our research, we reported significant improvements in both structural and functional outcomes after more than 1 year of follow-up, supporting faricimab's potential as a valuable treatment option.

Only a few authors have already explored the long-term changes in morpho-functional outcomes after faricimab therapy. Matsumoto et al. have reported one year results with an improvement in visual acuity and foveal thickness after three monthly injections followed by a TAE regimen [10]. Also Mukai et al., observed that 44% of patients maintained a 16-week treatment interval at the 1-year mark, similar to the 45.3% reported in the combined TENAYA and LUCERNE analyses [11]. Consistent with these results, at the most recent visit, 36.4% of our cohort was on a q16w regimen, and 36.4% on a q12w regimen, meaning that more than 70% of eyes were on extend dosing interval (at least q12w), similar to the data from TENAYA and LUCERNE [7].

A key finding of our research was the substantial reduction in CST, with the most pronounced decrease (−65 μm) occurring during the loading phase, and a successive stabilization suggesting effective disease control with the TAE regimen. Notably, eyes achieving a dry macula at week 16 (63.7%) required significantly fewer injections and maintained longer treatment intervals at the final visit, in line with previous reports underscoring the importance of achieving early and complete fluid resolution, which translates into reduced treatment burden for patients [10]. The observed reduction in both IRF (from 64% at V0 to 30% at V5) and SRF (from 82% at baseline to 21% at V5), along with the decrease in PED height, further reinforces the structural efficacy of faricimab. While a slight increase in PED height was noted during the TAE regimen, this change was not statistically significant and did not appear to negatively impact visual outcomes. Our results are consistent with those reported by Grimaldi et al., who showed improvements in SD-OCT parameters, such as macular volume (MV), CST and PED height, after 1 year of TAE regimen in treatment-naive eyes) [13].

In terms of functional outcomes, we observed a significant improvement in BCVA throughout the study. Over half of the treated eyes showed visual improvement, with 18.3% of eyes achieving a clinically meaningful gain of 0.3 LogMAR or more. Moreover, the mean visual improvement did not differ significantly between eyes with type 1 MNV and PCV, suggesting that faricimab is effective across different MNV subtypes. Interestingly, baseline CST, but not baseline BCVA, correlated with final visual outcomes, suggesting that the degree of initial retinal thickening may be a predictive factor for visual prognosis.

The effect of intravitreal anti-VEGF treatment on macular VD remains a topic of discussion. Early studies using the Retinal Vessel Analyzer (RVA) demonstrated significant vasoconstriction of retinal arterioles following ranibizumab injections, both in the short-term (7 and 30 days post-injection) and after one year. Studies using the Canon Laser Blood Flowmeter also reported reductions in arteriolar diameter, velocity, and blood flow after ranibizumab treatment [14–16]. On the other hand, studies employing OCTA have presented more nuanced findings. Cennamo et al. found no significant differences in VD after the loading phase of bevacizumab compared to baseline [17], while Resch et al. reported a decrease in VD of either the SCP and DCP after one year of anti-VEGF agents compared to age-matched controls [18]. Conversely, Lee et al. found that macular vascular density is more impacted by age itself, rather by the number of intravitreal anti-VEGF treatments [19].

Recently, Turksever et al. has observed a decrease in the density of SCP following anti-VEGF treatment, suggesting a possible vasoconstrictor effect of anti-VEGF therapy immediately after the first injection [20]. In our analysis, we have also noted an initial decrease in SCP, aligning with Turksever’s findings, which we attributed to this possible vasoconstrictor effects. However, this mechanism does not appear to be molecule-specific, but probably shared by all anti-VEGF treatments. Moreover, the VD of both plexi tended to stabilize during the follow-up. Consistent with the majority of published literature on this theme, we did not highlight a durable impact of faricimab on the pre-existing SCP and DCP architecture.

In comparison to pivotal trials that reported a low incidence of intraocular inflammatory adverse events (ranging from 0.2% to 2.5%) [6], we reported an excellent safety profile, with no documented cases of intraocular inflammation or adverse events. Notably, some authors highlighted a higher occurrence of RPE tears in real world studies compared to pivotal trials [7, 10], but this occurrence did not manifest in our cohort.

To the best of our knowledge, this is the first study to evaluate the macular structure and vasculature with OCT and OCT-angiography in treatment-naive nAMD after 1 year of faricimab. However, our study has several limitations. First, treatment decisions were based on the preferences of individual ophthalmologists, which could introduce bias into the selection process for faricimab injections. Second, the relatively small sample size and the lack of a control group limit the generalizability of our findings. Additionally, the follow-up period of 14 months, while providing valuable mid-term data, does not allow for assessment of long-term efficacy and safety. Future studies with larger sample sizes, longer follow-up periods, and a randomized controlled design are needed to confirm our findings and further elucidate the long-term effects of faricimab in nAMD.

In conclusion, our study demonstrates that faricimab is an effective and safe treatment option for treatment-naïve nAMD, leading to significant improvements in both structural and functional outcomes, even if the effect on microvascular architecture instead remains, to date, controversial. The ability to achieve extended treatment intervals in a substantial proportion of patients offers a potential advantage in terms of reduced treatment burden. Further research is warranted to confirm these findings and to evaluate the long-term efficacy and safety of faricimab in this patient population.

## Declarations

### Data sharing

The data that support the findings of this study are available from the corresponding author, MMC, upon reasonable request.
